# Dr. George McClellan

**Published:** 1913-05

**Authors:** 


Dr. George McClellan, Jefferson, 1870, the noted anatomist and surgeon, died at his home in Philadelphia, March 29, of heart disease, aged 64. He was a nephew of General McClellan. We had the pleasure of attending his clinics at the Blockley Hospital in 1888-9.

